# Metagenomic Insights into Ecophysiology of *Zetaproteobacteria* and *Gammaproteobacteria* in Shallow Zones within Deep-sea Massive Sulfide Deposits

**DOI:** 10.1264/jsme2.ME23104

**Published:** 2024-09-27

**Authors:** Nao Masuda, Shingo Kato, Moriya Ohkuma, Kazuyoshi Endo

**Affiliations:** 1 Department of Biological Sciences, Graduate School of Science, The University of Tokyo, 7–3–1 Hongo, Bunkyo-ku, Tokyo 113–0033, Japan; 2 Japan Collection of Microorganisms (JCM), RIKEN BioResource Research Center, 3–1–1 Koyadai, Tsukuba, Ibaraki 305–0074, Japan; 3 Submarine Resources Research Center (SRRC), Japan Agency for Marine-Earth Science and Technology (JAMSTEC), 2–15 Natsushima-cho, Yokosuka, Kanagawa 237–0061, Japan; 4 Department of Earth and Planetary Science, Graduate School of Science, The University of Tokyo, 7–3–1 Hongo, Bunkyo-ku, Tokyo 113–0033, Japan

**Keywords:** metagenomic ana­lysis, deep-sea hydrothermal sulfide deposits, yet-uncultivated microorganisms, *Zetaproteobacteria*, vertical distribution

## Abstract

Deep-sea massive sulfide deposits serve as energy sources for chemosynthetic ecosystems in dark, cold environments even after hydrothermal activity ceases. However, the vertical distribution of microbial communities within sulfide deposits along their depth from the seafloor as well as their ecological roles remain unclear. We herein conducted a culture-independent metagenomic ana­lysis of a core sample of massive sulfide deposits collected in a hydrothermally inactive field of the Southern Mariana Trough, Western Pacific, by drilling (sample depth: 0.52‍ ‍m below the seafloor). Based on the gene context of the metagenome-assembled genomes (MAGs) obtained, we showed the metabolic potential of as-yet-uncultivated microorganisms, particularly those unique to the shallow zone rich in iron hydroxides. Some members of *Gammaproteobacteria* have potential for the oxidation of reduced sulfur species (such as sulfide and thiosulfate) to sulfate coupled to nitrate reduction to ammonia and carbon fixation via the Calvin-Benson-Bassham (CBB) cycle, as the primary producers. The *Zetaproteobacteria* member has potential for iron oxidation coupled with microaerobic respiration. A comparative ana­lysis with previously reported metagenomes from deeper zones (~2‍ ‍m below the seafloor) of massive sulfide deposits revealed a difference in the relative abundance of each putative primary producer between the shallow and deep zones. Our results expand knowledge on the ecological potential of uncultivated microorganisms in deep-sea massive sulfide deposits and provide insights into the vertical distribution patterns of chemosynthetic ecosystems.

Deep-sea massive sulfide deposits are formed above and below hydrothermal fields down to hundreds of meters below the seafloor (mbsf) (Humphris *et al.*, 1995; Zierenberg *et al.*, 1998). These unique geological formations are created when metal ions (*e.g.*, Fe^2+^, Cu^2+^, and Zn^2+^) and hydrogen sulfide in hot fluid discharging from hydrothermal vents are cooled by cold seawater, leading to the precipitation of diverse metal sulfides, including pyrite (FeS_2_), pyrrhotite (Fe_1–x_S), sphalerite (ZnS), and chalcopyrite (CuFeS_2_) (Herzig and Hannington, 1995). Unlike many other places on Earth, the absence of sunlight and the presence of abundant reduced chemical species in these deep-sea environments lead to the development of unique chemosynthetic ecosystems (Orcutt *et al.*, 2020).

Abundant reduced sulfur species and ferrous iron in sulfide deposits serve as a fundamental energy source for chemosynthetic ecosystems (Wirsen *et al.*, 1993), even after the hydrothermal vent becomes inactive and abundant reduced chemical species in active hydrothermal fluid are no longer available for microorganisms. Previous studies on hydrothermally inactive sulfide chimneys (Suzuki *et al.*, 2004; Kato *et al.*, 2010; Sylvan *et al.*, 2012, 2013) or massive sulfide deposits buried below the seafloor (Kato *et al.*, 2015, 2018) suggested the presence of chemolithoautotrophic microorganisms that are distinct from those in active chimneys (*e.g.*, Zeng *et al.*, 2021; Zhou *et al.*, 2022a).

The global mass of sulfide deposits is estimated to be in the order of 10^8^ tons (Hannington *et al.*, 2011). They persist for several thousand years or longer after forming in hydrothermal fields (Lalou *et al.*, 1995; You and Bickle, 1998; Takamasa *et al.*, 2013). Hydrothermal systems likely existed on the early Earth (Russell and Hall, 1997; Martin *et al.*, 2008) and, thus, sulfide deposits must also have been present (Vearncombe *et al.*, 1995; Runge *et al.*, 2023). The prevalence of these sulfide deposits on the seafloor underscores their potential significance in global biogeochemical cycles throughout the Earth’s history. Despite the importance of these unique ecosystems, deep-sea massive sulfide deposits remain one of the least explored habitats on Earth.

Massive sulfide deposits have been found at two deep-sea hydrothermal vent fields (called the Pika and Archaean sites) in the Southern Mariana Trough, Western Pacific (Nakamura *et al.*, 2015). The mineral and microbial community compositions of sulfide deposits have been shown to vary with depth beneath the seafloor (Kato *et al.*, 2015, 2018; Nakamura *et al.*, 2015). In drilling core samples from the Archaean site, of which the major component was FeS_2_, at depths of 1.86–1.96 mbsf, uncultivated members of *Nitrospirota* and *Desulfobacterota* predominated as shown by a 16S rRNA gene ana­lysis (Kato *et al.*, 2015). Their metabolic potential was demonstrated by a metagenomic ana­lysis (Kato *et al.*, 2018). In contrast, iron hydroxides were observed on the fracture surface of veins in another core sample collected from the Pika site at 0.52 mbsf (Nakamura *et al.*, 2015). The formation of these iron hydroxides occurs in the presence of oxygen in penetrating seawater, implying the existence of a different ecosystem associated with constraints in elemental cycling. Uncultivated members of‍ ‍*Gammaproteobacteria* were abundant and those of *Zetaproteobacteria* were only detected in a shallower core sample (Kato *et al.*, 2015). However, the metabolic potential of uncultivated members remains unknown.

In the present study, we conducted a metagenomic ana­lysis of iron hydroxides of a sulfide core sample collected at a depth of 0.52 mbsf (Fig. 1) to infer the ecology of microbial communities and biogeochemical cycling in oxygenated subseafloor environments within massive sulfide deposits. We successfully obtained metagenome-assembled genomes (MAGs) and reconstructing their metabolic pathways. We herein focused on the metabolic potential of as-yet-uncultivated chemolithoautotrophs associated with the cycles of iron and sulfur and discussed their ecological roles.

## Materials and Methods

### Sample collection and DNA extraction

**Fig. 1.**
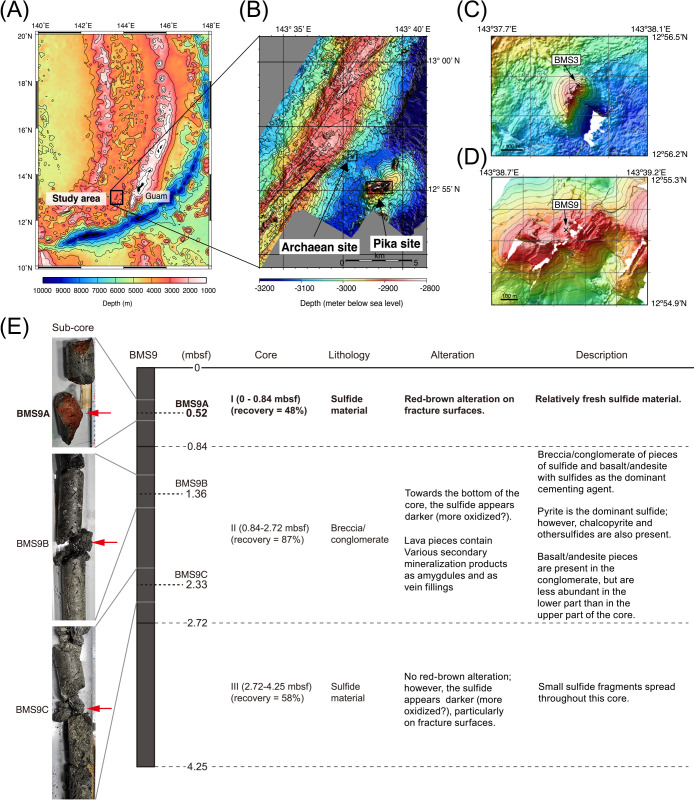
Bathymetry map of sampling sites and visual core description of the core sample BMS9. (A) General location and (B) close-up map of the Southern Mariana Trough, and the drilling points of (C) BMS3 at the Archaean site and (D) BMS9 at the Pika site adapted from Kato *et al.* (2015). (E) Visual core description and photos of the core sample BMS9 modified from Kato *et al.* (2015). Red-brown oxides are observed on the fractured surface of the sub-sample BMS9A.

The sample collection and DNA extraction procedures of sub-seafloor sulfide deposits were previously described (Kato *et al.*, 2015). In brief, a sulfide core sample (BMS9) was collected from a deep-sea hydrothermal vent field at the Pika site (Nakamura *et al.*, 2015) (12°55.14′N, 143°38.93′E, water depth of 2,804 m) in the Southern Mariana Trough using a benthic multi-coring system (BMS) (Marumo *et al.*, 2008) (Fig. 1). Core sampling was conducted during the TAIGA10M cruise of the R/V Hakurei-Maru No. 2 (JOGMEG, Japan) in June 2010. The core sample was stored at −‍80°C until DNA extraction. DNA was extracted from portions of the sub-core sample BMS9A, obtained from part of the core sample BMS9 corresponding to a depth of 0.52 mbsf.

### Metagenomic sequencing and MAG construction

In this study, a portion of the same DNA extracts previously subjected to PCR amplicon ana­lyses (Kato *et al.*, 2015) was used for shotgun library construction with a KAPA Hyper Prep Kit for Illumina (KAPA Biosystems). The metagenomic library was sequenced on an Illumina MiSeq platform (MiSeq PE300). The trimming and filtering of raw reads were performed using the read_qc module of metaWRAP version 1.3.2 (Uritskiy *et al.*, 2018). High-quality reads were then assembled into contigs using SPAdes version 3.14.1 (Bankevich *et al.*, 2012) with the options ‘--meta -k 77, 99, 111, 121’. As a result, 141,910 contigs of 1,000‍ ‍bp or longer (total length of 310,292,534‍ ‍bp, N50 of 2,274‍ ‍bp) were obtained. A total of 76.25% of all high-quality reads were mapped on the contigs. Subsequently, 18,511 contigs of 3,000‍ ‍bp or longer (total length of 123,711,640‍ ‍bp, N50 of 7,168‍ ‍bp) were used in further ana­lyses. They were firstly binned using the binning module of metaWRAP, including MetaBAT2 (Kang *et al.*, 2015), MaxBin2 (Wu *et al.*, 2016), and CONCOCT (Alneberg *et al.*, 2014), and the three bin sets were refined using the bin_refinement module of metaWRAP. In addition, contigs were separately binned using Vamb version 3.0.3 (Nissen *et al.*, 2021) and MetaCoAG version 1.1.0 (Mallawaarachchi and Lin, 2022). The three bin sets from the bin_refinement module (1st run), Vamb, and MetaCoAG were then refined using the bin_refinement module, resulting in 17 MAGs (>70% completeness, <5% contamination). The quality of MAGs was checked by CheckM2 version 0.1.3 (Chklovski *et al.*, 2023). Based on genome reporting standards (Bowers *et al.*, 2017), all MAGs reported in the present study were medium- or high-quality drafts. MAGs were annotated using DFAST version 1.2.19 (Tanizawa *et al.*, 2018) with Prodigal version 2.6.3 (Hyatt *et al.*, 2010) for the prediction of protein-coding regions (CDSs), tRNAscan-SE (Chan *et al.*, 2021) for the identification of tRNA genes, and Barrnap (https://github.com/tseemann/barrnap) for the identification of rRNA genes.

### Taxonomic profiling

Taxonomic profiling of the whole metagenome was performed with high-quality reads using SingleM version 0.15.0 (https://github.com/wwood/singlem) for two highly conserved marker genes (*i.e.*, *rpsB* and *rplB*) and phyloFlash version 3.4 (Gruber-Vodicka *et al.*, 2020) for the 16S rRNA gene. The relative abundance of each taxonomic clade in publicly released metagenomes was analyzed using Sandpiper version 0.1.1 (https://sandpiper.qut.edu.au) screening 248,666 metagenomes deposited in the NCBI SRA database by SingleM. The taxonomic classification of MAGs was performed using Genome Taxonomy Database (GTDB)-tk version 2.3.0 (Chaumeil *et al.*, 2019; Parks *et al.*, 2022) with the R214 database.

### Phylogenetic ana­lysis

To construct a phylogenomic tree for MAGs, the concatenated alignment of 120 marker proteins provided by GTDB-tk was trimmed using TrimAl with the “-automated1” option (Capella-Gutiérrez *et al.*, 2009) and used to contrast a maximum likelihood (ML) tree using IQ-TREE version 2.2.0 with the LG+I+G4 model (Minh *et al.*, 2020). To construct a phylogenetic tree of 16S rRNA genes for *Zetaproteobacteria*, nucleotide sequences deposited at the ZetaHunter database (McAllister *et al.*, 2018) and obtained from *Zetaproteobacteria* MAGs in this study were aligned using MAFFT v.7.490 (Katoh and Standley, 2013). The alignment was trimmed using TrimAl with the ‘-automated1’ option. An ML tree was constructed using IQ-TREE with the GTR+F+I+G model. Ultrafast bootstrap support values were computed with 1,000 replicates for all trees.

### Metabolic prediction

Genes involved in carbon, nitrogen, and sulfur metabolism were surveyed in MAGs using METABOLIC version 4.0 (Zhou *et al.*, 2022b). In addition, genes involved in sulfide oxidation or sulfate reduction, *i.e.*, *dsrABCDEFHJKMOP*, *aprAB*, and *sat*, were annotated using DiSCo version 1.0.0 (Neukirchen and Sousa, 2021). Genes involved in iron oxidation, such as *cyc1* and *cyc2*, were annotated using FeGenie version 1.0 (Garber *et al.*, 2020).

### Data availability

The raw sequence data produced by metagenomic shotgun sequencing were deposited into the DNA Data Bank of Japan (DDBJ) under the BioProject accession number PRJDB5792 and the DDBJ Sequence Read Archive (DRA) accession number DRA017006. The nucleotide sequences of the MAGs of BMS9Abin02, BMS9Abin05, BMS9Abin07, BMS9Abin11, BMS9Abin12, BMS9Abin13, BMS9Abin15, BMS9Abin17, BMS9Abin18, BMS9Abin23, BMS9Abin25, BMS9Abin26, BMS9Abin28, BMS9Abin29, BMS9Abin34, BMS9Abin36, and BMS9Abin37 were deposited into DDBJ under accession numbers BTQB01000000, BTQE01000000, BTQG01000000, BTQK01000000, BTQL01000000, BTQM01000000, BTQO01000000, BTQP01000000, BTQQ01000000, BTQV01000000, BTQX01000000, BTQY01000000, BTQZ01000000, BTRA01000000, BTRF01000000, BTRG01000000, and BTRH01000000, respectively.

## Results and Discussion

### Microbial community structures

**Fig. 2.**
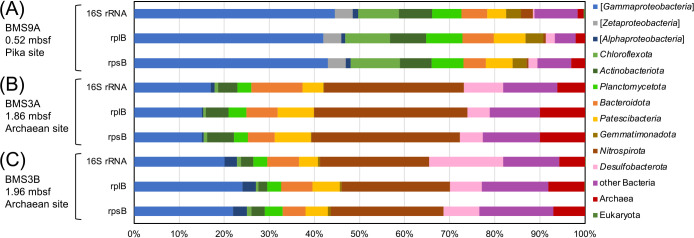
Microbial community structures of sulfide core samples. Relative abundance of each taxon in the whole community of (A) BMS9A, (B) BMS3A, and (C) BMS3B based on 16S rRNA genes and *rplB* and *rpsB*. Data for BMS3A and BMS3B from Kato *et al.* (2018) were re-analyzed in this study.

To assess the microbial community structure in the BMS9A metagenome of the shallow zone with iron oxides within massive sulfide deposits at the Pika site, we conducted ana­lyses of two highly conserved single-copy marker genes (*i.e.*, *rpsB* and *rplB*) in addition to the ana­lysis of 16S rRNA genes present in metagenomic reads (Fig. 2A and S1). Overall, the dominance of *Gammaproteobacteria* and the detection of *Zetaproteobacteria* were consistent with the previous findings of the 16S rRNA gene amplicon ana­lysis (Kato *et al.*, 2015). In addition, we re-analyzed the BMS3A and BMS3B metagenomes of the deep zones at the Archaean site (Kato *et al.*, 2018) (Fig. 2B, 2C, and S1). The relative abundance of each taxon within *Gammaproteobacteria* is shown in Fig. S2. As reported by Kato *et al.* (2015), the community of BMS9A differed from those of the deeper sub-seafloor sulfide deposit samples, which may reflect the effects of oxygenated conditions on both the microbial community and mineralogy (Fig. 1; Table S1 and S2). Furthermore, in this community, archaeal members were not abundant (~2% of all reads), which is also consistent with the findings of the quantitative PCR ana­lysis (Kato *et al.*, 2015).

### Genome-centric metagenomics

**Fig. 3.**
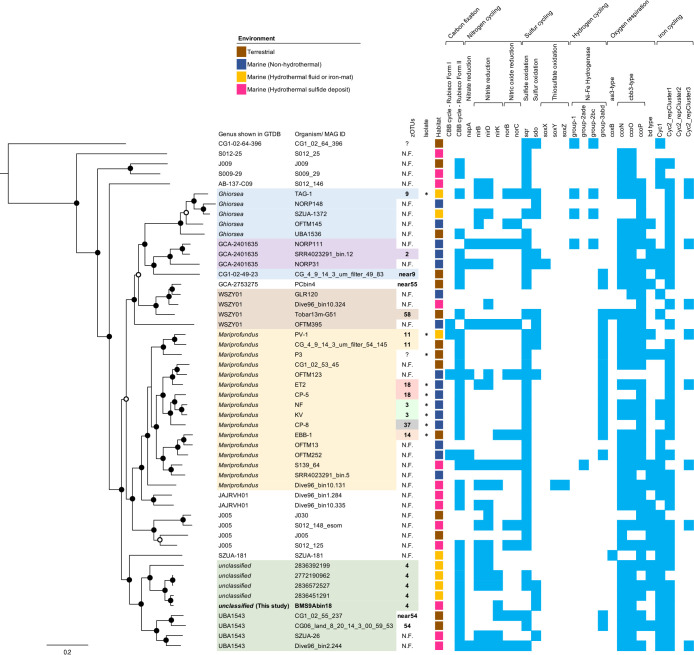
Phylogeny and gene contents of *Zetaproteobacteria* MAGs. A maximum-likelihood tree of 120 concatenated bacterial single-copy marker proteins of *Zetaproteobacteria* MAGs is shown on the left side. Nodes with ultrafast bootstrap support values higher than 90 and 50% are shown with filled and open circles, respectively. The scale bar shows 0.2 substitutions per site. A heat map showing the presence (light blue) or absence (white) of genes involved in carbon, nitrogen, sulfur, hydrogen, oxygen, and iron metabolism is shown on the right side. zOTU numbers are also shown. ‘?’ indicates that zOTU numbers were not clearly identified. N.F. indicates that 16S rRNA was not found in MAGs. ‘*’ indicates an isolated cultivate. The habitats of each MAG are categorized into four groups. The sequence of *Magnetococcus marinus* (CP000471) was used as the outgroup (not shown).

**Fig. 4.**
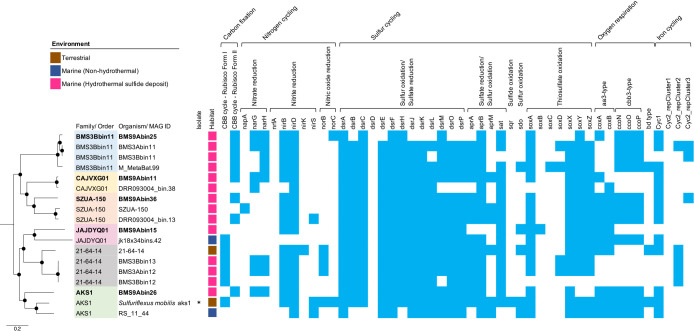
Phylogeny and gene contents of *Gammaproteobacteria* MAGs. A maximum-likelihood tree of 120 concatenated bacterial single-copy marker proteins of *Gammaproteobacteria* MAGs is shown on the left side. Nodes with ultrafast bootstrap support values higher than 90% are shown with filled circles. The scale bar shows 0.2 substitutions per site. A heat map showing the presence (light blue) or absence (white) of genes involved in carbon, nitrogen, sulfur, oxygen, and iron metabolism is given on the right side. ‘*’ indicates an isolated cultivate. The habitats of each MAG are categorized into three groups. The sequence of *Magnetococcus marinus* (CP000471) was used as the outgroup (not shown).

We obtained 17 MAGs (>70% completeness, <5% contamination) from the BMS9A sample (Table S1), including 3 MAGs with high genome completeness (>90%) with low contamination (<0.8%). Each MAG was identified as a cluster based on contig lengths, the GC content of contigs (%), and the mean coverage of contigs (Fig. S3; Table S1). Based on the GTDB classification, these MAGs were affiliated with the phyla *Acidobacteriota*, *Actinomycetota*, *Bacteroidota*, *Chloroflexota*, *Desulfobacterota*, *Gemmatimonadota*, *Patescibacteria*, and *Pseudomonadota*. Genes involved in major metabolic pathways for each MAG are summarized in Table S1. Since sulfide deposits contain both ferrous iron and reduced sulfur species, which serve as energy sources for chemolithoautotrophs, further ana­lyses of MAGs assigned to the classes *Zetaproteobacteria* (one MAG) and *Gammaproteobacteria* (five MAGs) of *Pseudomonadota* were conducted. Since the relative frequencies of these members were high (Table S1), they were potential primary producers in the ecosystem. The gene encoding Form II ribulose-1,5-bisphosphate carboxylase/oxygenase (RubisCO), which is a key enzyme for carbon fixation via the Calvin-Benson-Bassham (CBB) cycle under microaerobic conditions (Badger and Bek, 2008), was found in most *Zetaproteobacteria* and *Gammaproteobacteria* MAGs (Fig. 3 and 4; Table S1 and S3). Microaerophilic isolates of *Zetaproteobacteria* typically possess Form II RubisCO (Singer *et al.*, 2011; Fullerton *et al.*, 2017; Mori *et al.*, 2017; Blackwell *et al.*, 2020). In addition, genes encoding *aa3*- or *cbb3*-type cytochrome *c* oxidase for oxygen respiration, which adapt to high or low O_2_ conditions (Pitcher and Watmough, 2004; Osamura *et al.*, 2017; McAllister *et al.*, 2019, 2020), were detected in most *Gammaproteobacteria* and *Zetaproteobacteria* MAGs, suggesting their growth under (micro)aerobic conditions. This contrasts with the previously reported MAGs of dominant microorganisms in deeper samples, such as members of *Nitrospirota* and *Desulfobacterota* (Fig. 2B and C), which lack these oxygen respiratory genes (Kato *et al.*, 2018), highlighting the specificity of this shallow sample.

### Zetaproteobacteria

Based on the phylogenetic ana­lysis of the 16S rRNA gene with the ZetaOTU classification (McAllister *et al.*, 2018), the *Zetaproteobacteria* MAG (BMS9Abin18) obtained in the present study was classified into zOTU4 (Fig. S4). 16S rRNA genes in zOTU4 have been detected in various hydrothermal environments (Dang *et al.*, 2008; Forget *et al.*, 2010; McAllister *et al.*, 2011; Kato *et al.*, 2015; Hassenrück *et al.*, 2016; Fullerton *et al.*, 2017; Hager *et al.*, 2017; Scott‍ ‍*et al.*, 2017). The 16S rRNA gene in the MAG BMS9Abin18 showed 99.78% similarity to the environmental clone BMS9AB48 (NCBI accession no. AB722105) that we previously reported from the same sulfide core sample by 16S rRNA gene amplicon sequencing (Kato *et al.*, 2015). Therefore, we successfully reconstructing a MAG with an almost identical 16S rRNA sequence to the environmental clone BMS9AB48, which allowed us to investigate the metabolic potential of the uncultivated zetaproteobacterial member in the massive sulfide deposit. The gene list of BMS9Abin18 is shown in Table S3.

We herein analyzed 50 representative *Zetaproteobacteria* MAGs in the GTDB (Release 08-RS214) and the Joint Genome Institute (JGI) Integrated Microbial Genomes and Microbiomes (IMG/M) database with BMS9Abin18 (Fig. 3; Table S4). Based on the GTDB taxonomy classification, the‍ ‍clade containing BMS9Abin18 and four MAGs from marine hydrothermal iron-rich mats (Fullerton *et al.*, 2017; McAllister *et al.*, 2020) represented a genus-level clade within *Zetaproteobacteria* (Table S1), which was distinguished from the closely-related genus-level clade “g__UBA1543” (Fig. 3). Most *Zetaproteobacteria* MAGs, including BMS9Abin18, had the gene for FormII RuBisCO (Fig. 3) as previously reported (McAllister *et al.*, 2019, 2020). Regarding the microaerobic iron oxidation pathway (McAllister *et al.*, 2019, 2020), BMS9Abin18 and most of the other MAGs encoded genes for Cyc2, Cyc1, and *cbb3-*type cytochrome c oxidase, whereas only a few of them encoded the gene for *aa3*-type cytochrome c oxidase, which adapts to higher O_2_ conditions than *cbb3-*type cytochrome c oxidase (Pitcher and Watmough, 2004; Osamura *et al.*, 2017; McAllister *et al.*, 2019, 2020). We also found that most *cyc2* belonged to Cluster 1, with some belonging to Cluster 3. Both clusters are known to be involved in iron oxidation (Jeans *et al.*, 2008; McAllister *et al.*, 2020; Keffer *et al.*, 2021). As previously proposed (Barco *et al.*, 2015; McAllister *et al.*, 2019, 2020), an electron from Fe(II) likely passes through Cyc2 and then to Cyc1 or another electron carrier, followed by cytochrome *c* oxidase and finally to O_2_, generating proton motive force. No genes for hydrogenases were found in BMS9Abin18, although some MAGs or genomes of the hydrogen-oxidizing *Ghiorsea* spp. (Mori *et al.*, 2017) of *Zetaproteobacteria* had these genes (Fig. 3). In addition, BMS9Abin18 encoded *nirK* for nitrite reductase, which reduces nitrite to nitric oxide as an intermediate step in denitrification (Kobayashi *et al.*, 2018). Two of the four MAGs classified in the same genus-level clade of BMS9Abin18 and three of the four MAGs in the closely related genus-level clade “g__UBA1543” also encoded *nirK*, suggesting that members of these clades commonly mediate nitrite reduction. However, it remains unclear whether iron oxidation may be coupled with nitrite reduction. In summary, the uncultivated zetaproteobacterial member derived from BMS9Abin18 appeared to be a primary producer in the shallow zone of the massive sulfide deposit, which was rich in iron hydroxides, fixing CO_2_ via the CBB cycle and retrieving energy by iron oxidation coupled with microaerobic respiration.

### Gammaproteobacteria

The five MAGs of *Gammaproteobacteria* recovered in‍ ‍the present study were classified into the orders *Acidiferrobacterales* (BMS9Abin11 and BMS9Abin36) and‍ ‍*Arenicellales* (BMS9Abin25), and order-level clades of‍ ‍“o__AKS1” (BMS9Abin26) and “o__JAJDYQ01” (BMS9Abin15) (Fig. 4; Table S5). The MAGs of *Acidiferrobacterales* were classified into the family-level clades of “f__CAJVXG01” (BMS9Abin11) and “f__SZUA-150” (BMS9Abin36). The BMS9Abin25 of *Arenicellales* were classified in the family-level clade of “f__BMS3Bbin11”. Among the above order- or family-level clades, only “o__AKS1” contained a cultivated isolate, *i.e.*, the chemolithoautotrophic sulfur-oxidizing bacterium *Sulfuriflexus mobilis* (Kojima and Fukui, 2016). Despite the vast number of genomes (>400,000) in the latest GTDB, only a few MAGs were affiliated in each family-level clade‍ ‍of “f__CAJVXG01”, “f__BMS3Bbin11”, or “f__SZUA-150”, all of which were recovered from deep-sea hydrothermal sulfide deposits (Fig. 4; Table S5) (Kato *et al.*, 2018; Wang *et al.*, 2018; Hou *et al.*, 2020). A read-based relative abundance ana­lysis using Sandpiper indicated that members of these three families were the most abundant in the metagenomes of deep-sea hydrothermal sulfide deposits (Table S6). Therefore, members of “f__CAJVXG01”, “f__BMS3Bbin11”, and “f__SZUA-150” appear to preferably thrive in deep-sea hydrothermal sulfide deposits. Two (“f__CAJVXG01” and “f__BMS3Bbin11”) of the three families were also relatively abundant in the metagenome of our shallow sulfide core sample (Fig. S2A).

**Fig. 5.**
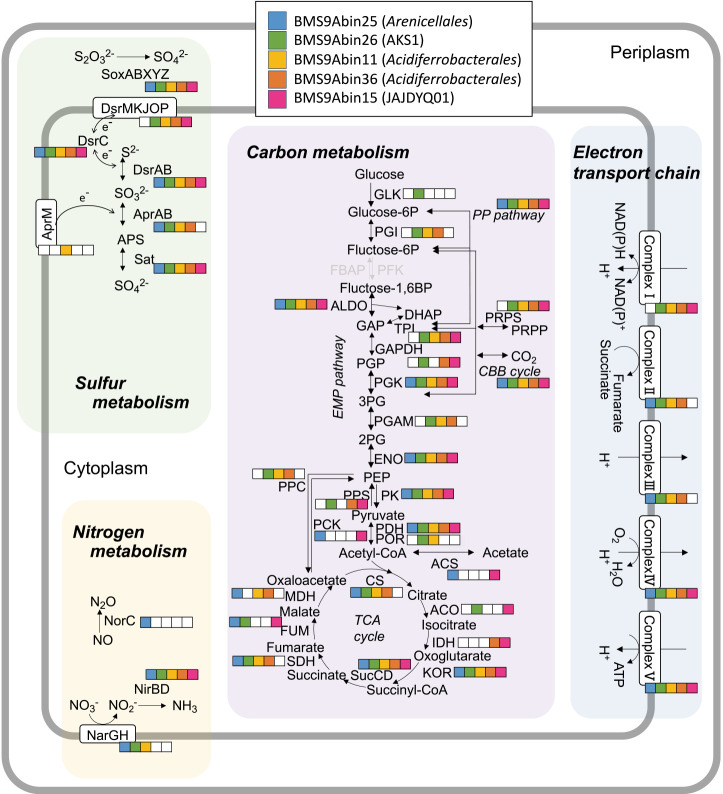
Metabolic potential of five *Gammaproteobacteria* MAGs obtained in this study. Details of genes in the pathways are listed in Table S3. Colored squares (blue, green, yellow, orange, and pink) indicate genes encoded in the MAGs of BMS9Abin25 (f__BMSBbin11), BMS9Abin26 (f__AKS1), BMS9Abin11 (f__CAJVXG01), BMS9Abin36 (f__SZUA-150), and BMS9Abin15 (f__JAJDYQ01), respectively. The pathways with genes not found in any MAGs were colored in gray.

The metabolic capacities of the five *Gammaproteobacteria* MAGs and their closely related MAGs are shown in Fig. 4 and 5 and Table S3. Although MAGs differed at the family or order level, their metabolic potentials were similar. Most had genes for the Sox system (*soxABXYZ*; thiosulfate disproportionation to sulfate and elemental sulfur [Berben *et al.*, 2019]), the oxidative Dsr-Apr-Sat system (*i.e.*, the reversal of the dissimilatory sulfate reduction pathway [Pott and Dahl, 1998; Meyer and Kuever, 2007]), and nitrite reductase (NirBD), which is involved in dissimilatory nitrate reduction to ammonium. Some MAGs‍ ‍in the family-level clades “f__BMS3Bbin11” and “f__CAJVXG01” and BMS9Abin26 also encode genes for membrane-bound nitrate reductase (NarGH). As described above, all of our *Gammaproteobacteria* MAGs encoded some genes for the *aa3*- and/or *cbb3-*type cytochrome c oxidase subunits of (micro)aerobic respiration and for Form II RuBisCO. Therefore, they may be facultative anaerobic chemolithoautotrophs capable of sulfur oxidation coupled with nitrate reduction, and may further reduce nitrite to ammonia. Since some of the MAGs encoded *cyc1* or Cluster 2/3 *cyc2*, some gammaproteobacterial members may be able to oxidize Fe^2+^ as an energy source.

Kato *et al.* (2018) previously reported that gamma­proteobacterial members of “f__BMS3Bbin11” and “g__BMS3Bbin12” of “f__21-64-14” predominated in deeper zones (1.86 and 1.96 mbsf) within the massive sulfide deposits at another hydrothermal site of the Southern Mariana Trough (Fig. S2B and C; Table S2). In the present study, we recovered the MAGs of “f__BMS3Bbin11” from the shallow zone, but not those of “g__BMS3Bbin12”. The relative abundance of “g__BMS3Bbin12” in the shallow metagenome was very low (<0.1%) (Fig. 2A and S2A). It is important to note that no genes for oxygen respiration were found in the MAG BMS3Abin12 from the deep zone and its close relatives of “g__BMS3Bbin12” (Fig. 4). Therefore, the members of “g__BMS3Bbin12” may be obligate anaerobes and, thus, only predominate in the deeper anoxic zones of massive sulfide deposits.

### Vertical distribution patterns of chemolithoautotrophs within massive sulfide deposits

The previous 16S rRNA gene-based ana­lysis showed the vertical distribution of diverse uncultivated microorganisms within massive sulfide deposits at the two deep-sea hydrothermal vent sites (Pika and Archaean sites) in the Southern Mariana Trough (Kato *et al.*, 2015); however, their metabolic capabilities remain unclear. To date, the MAGs of these microorganisms, including putative chemolithoautotrophs, were obtained only from metagenomes in the deeper zones (1.86 and 1.96 mbsf) at the Archaean site (Table S2) (Kato *et al.*, 2018). In the present study, we obtained MAGs from the shallower zone (0.52 mbsf) of massive sulfide deposits at the Pika site, in which orange iron (hydr)oxide minerals were rich, suggesting oxic seawater penetration. These iron (hydr)oxide minerals were not observed in sulfide core samples of the deeper zones at the Archaean site.

**Fig. 6.**
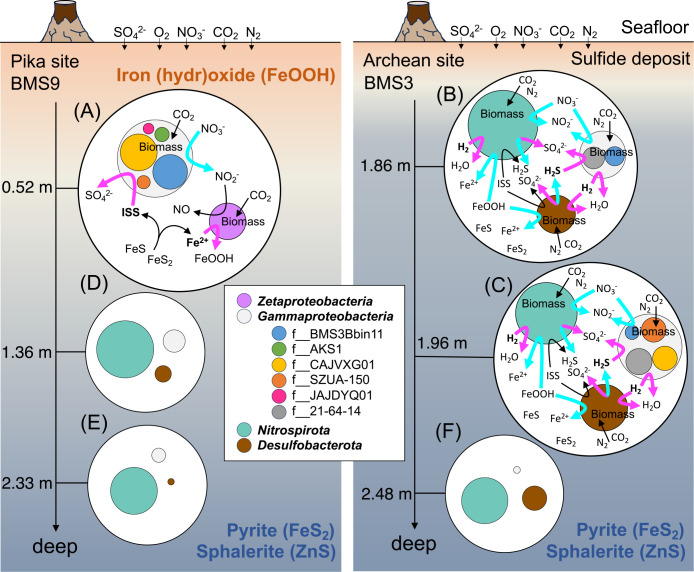
A model of biogeochemical cycling by major chemolithoautotrophs within massive sulfide deposits below the seafloor. (A) The proposed model in this study. (B and C) The previously reported model based on metagenomics (10). (D, E, and F) Abundant taxa based on the previous 16S rRNA gene amplicon ana­lysis (11). Their metabolic potential is still unclear. The colored arrows indicate redox reactions (magenta, oxidation; sky-blue, reduction). The size of the circle for each taxon roughly indicates the degree of abundance.

Based on the metabolic potential of putative chemolithoautotrophs, we propose a model for the biogeochemical cycling of carbon, sulfur, nitrogen, and iron within massive‍ ‍sulfide deposits (Fig. 6). In the shallower zone‍ ‍at‍ ‍the Pika site, abundant members in the family-level‍ ‍clades “f__CAJVXG01” and “f__BMS3Bbin11” of *Gammaproteobacteria* appeared to gain energy by the oxidation of S^2–^ and S_2_O_3_^2–^ to SO_4_^2–^ coupled with the reduction of O_2_ or NO_3_^–^, and fixed CO_2_ into the biomass. The detected member of *Zetaproteobacteria* appeared to gain energy by the oxidation of Fe^2+^ to Fe^3+^ and the reduction of O_2_. In this environment without hydrothermal fluid discharging, the energy sources of Fe^2+^ and reduced sulfur species may be produced by the abiotic weathering reactions of surrounding iron sulfides (FeS and FeS_2_) and by the microbial reduction of SO_4_^2–^ and Fe^3+^ in deeper zones.

In contrast, in deeper zones at the Archaean site, the dominant members of *Nitrospirota* and *Desulfobacterota* appeared to be strictly anaerobic, gained energy by the oxidation of H_2_S and H_2_ coupled with the reduction of NO_3_^–^, SO_4_^2–^, and Fe^3+^, and fixed CO_2_ into the biomass as previously reported (Kato *et al.*, 2018). Although members of *Gammaproteobacteria* were partially common in shallow and deep zones at the two sites, the putative strict anaerobic members in the family-level clade “f__21-64-14” were only abundant in the deeper zone. The observed difference in abundant members between the shallow and deep zones may reflect oxygen availability within massive sulfide deposits, which is consistent with the observed difference in the mineralogy of collected samples (Table S2).

In summary, we showed the metabolic potential of as-yet-uncultured members of *Zetaproteobacteria* and *Gammaproteobacteria* inhabiting shallow zones rich in iron oxides within massive sulfide deposits of the Southern Mariana Trough. Both members appear to function as chemolithoautotrophs that are capable of oxidizing iron and sulfur as their energy sources, respectively. Since they are more likely to inhabit metal sulfide surfaces rather than being free-living, simulating these conditions in experiments may enable us to cultivate these as-yet-uncultivated microorganisms and verify their metabolic capabilities in the future. We also revealed the previously unknown vertical distribution patterns of chemosynthetic ecosystems within massive sulfide deposits in the Southern Mariana Trough, which are independent of effects from active hydrothermal fluid flow (*e.g.*, temperature and the availability of electron donors, such as H_2_ and CH_4_) and may simply be controlled by the availability of electron acceptors (*e.g.*, O_2_, NO_3_^–^, and SO_4_^2–^) in penetrating seawater. Although no metagenomic data on subseafloor sulfide samples are available, except for the Southern Mariana Trough hydrothermal fields, specific members of *Gammaproteobacteria*, *Nitrospirota*, and *Desulfobacterota*, which were identified as putative primary producers in Southern Mariana Trough sulfide core samples, have been abundantly detected in deep-sea sulfide deposits (Suzuki *et al.*, 2004; Kato *et al.*, 2010; Sylvan *et al.*, 2012; Li *et al.*, 2017; Meier *et al.*, 2019; Hou *et al.*, 2020; Zhou *et al.*, 2022a). Accordingly, we hypothesize that the distribution patterns of chemosynthetic ecosystems are globally similar among massive sulfide deposits in marine hydrothermal fields. Further efforts regarding subseafloor drilling and microbiological ana­lyses of massive sulfide deposits in various areas are needed to evaluate this hypothesis, which will provide a more detailed understanding of the significance of microorganisms within massive sulfide deposits in global biogeochemical cycles.

## Citation

Masuda, N., Kato, S., Ohkuma, M., and Endo, K. (2024) Metagenomic Insights into Ecophysiology of *Zetaproteobacteria* and *Gammaproteobacteria* in Shallow Zones within Deep-sea Massive Sulfide Deposits. *Microbes Environ ***39**: ME23104.

https://doi.org/10.1264/jsme2.ME23104

## Supplementary Material

Supplementary Material 1

Supplementary Material 2

Supplementary Material 3

Supplementary Material 4

Supplementary Material 5

Supplementary Material 6

Supplementary Material 7
