# Supplementary material for: Metagenomic Insights into Ecophysiology of Zetaproteobacteria and Gammaproteobacteria in Shallow Zones within Deep-sea Massive Sulfide Deposits
Source: Microbes Environ. 2024 Sep 27;39(3):ME23104. doi: 10.1264/jsme2.ME23104 (PMC11427306; doi:10.1264/jsme2.ME23104)
Supplement: Supplementary file 1 — Supplementary Material 1 [file 39_23104_s1.pdf]

# Supplementary Materials

**Fig. S1. Microbial community structures of the sulfide core samples**. Relative abundance of each taxon in the whole community of BMS9A based on (A) 16S rRNA genes and (B) *rplB* and *rpsB*. Relative abundance of each taxon in the whole community of (C) BMS3A and (D) BMS3B based on *rplB* and *rpsB*. Data for BMS3A and BMS3B from Kato *et al*. (2018) was re-analyzed in this study.

**Fig. S2. Population structures of *Gammaproteobacteria*.** Relative abundance of each taxon within *Gammaproteobacteria* of (A) BMS9A, (B) BMS3A, and (C) BMS3B based on *rplB* and *rpsB*. Data for BMS3A and BMS3B from Kato *et al*. (2018) was re-analyzed in this study. Colored squares (blue, green, yellow, orange, pink, and grey) indicate family-level clade of the MAGs of BMS9Abin25 (f__BMSBbin11), BMS9Abin26 (f__AKS1), BMS9Abin11 (f__CAJVXG01), BMS9Abin36 (f__SZUA-150), BMS9Abin15 (f__JAJDYQ01), and BMS3Bbin12 (f__21-64-14), respectively.

**Fig. S3**. **The MAG clusters of this study.** Each MAG can be identified as a cluster based on contig lengths (size of dots), GC content of contigs (%), and mean coverage of contig.

**Fig. S4. Phylogenetic tree of 16S rRNA genes of *Zetaproteobacteria*.** 16S rRNA genes of *Zetaproteobacteria* MAGs were aligned with those downloaded from the ZetaHunter database (41), and the maximum-likelihood tree was constructed. The sequence of *Magnetococcus marinus* (CP000471) was used as the out-group. Ultrafast bootstraps are shown, and the scale bar shows 0.06 substitutions per site. The 16S rRNA gene sequences from ZetaHunter database have been identified with their corresponding zOTU numbers. Comparing with them, 16S rRNA gene of *Zetaproteobacteria* MAGs were assigned to each zOTU number. '?' indicates the zOTU number were not clearly identified in the 16S rRNA gene tree.

**Table S1. Summary of MAGs reconstructed from the BMS9A metagenome.**

**Table S2. Summary of the sulfide core samples used in the present and previous studies.**

**Table S3. List of genes of *Gammaproteobacteria* and *Zetaproteobacteria* MAGs.**

**Table S4. Summary of *Zetaproteobacteria* MAGs.**

**Table S5. Summary of *Gammaproteobacteria* MAGs.**

**Table S6. Relative abundance of the family-level clades of *Gammaproteobacteria* in the publicly released metagenomes.**

**Fig. S1**

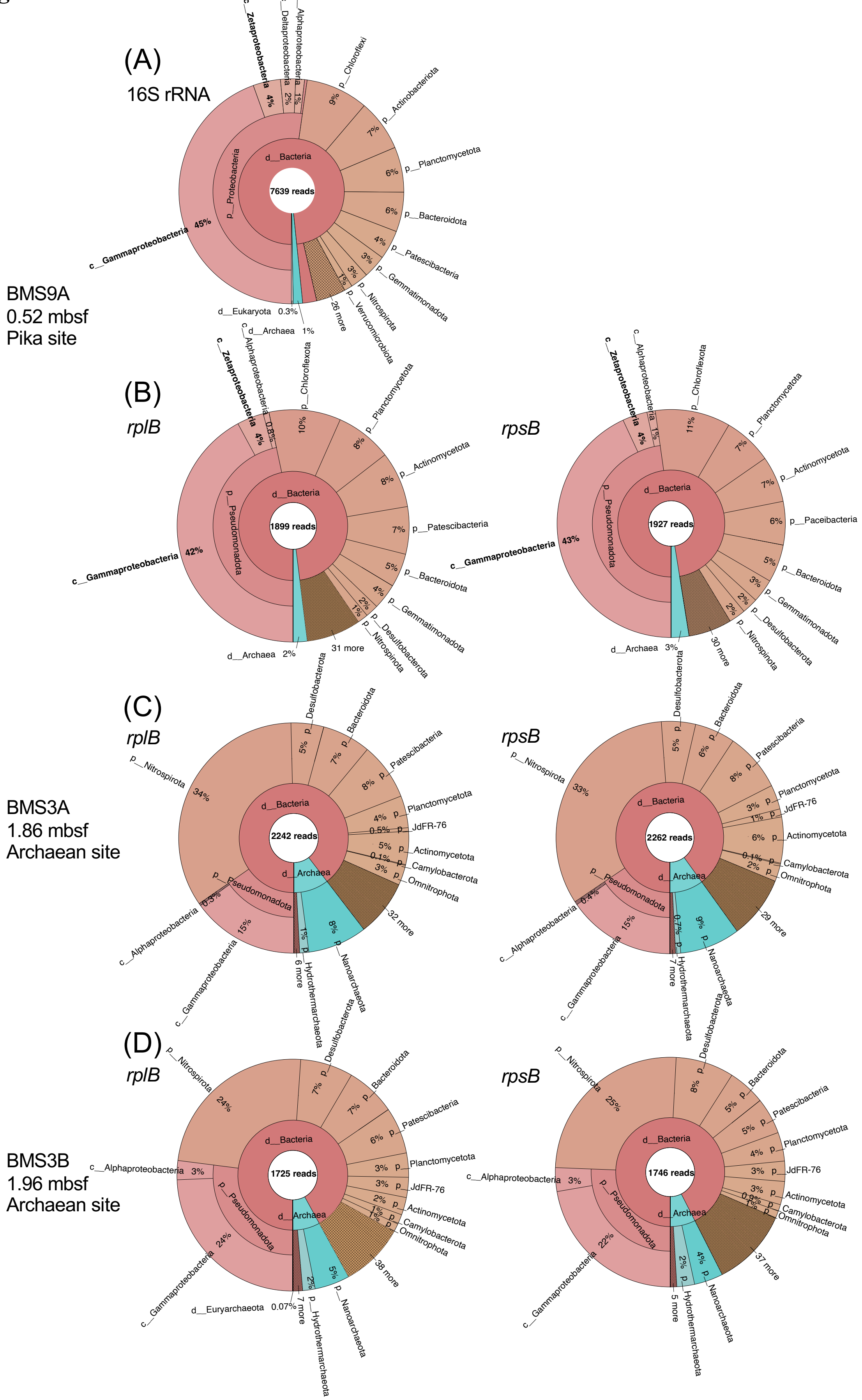

(A)
16S rRNA
BMS9A
0.52 mbsf
Pika site
7639 reads
d__Bacteria
p__Proteobacteria
c__Gammaproteobacteria 45%
c__Zetaproteobacteria 4%
c__Deltaproteobacteria 2%
c__Alphaproteobacteria 1%
p__Chloroflexi 9%
p__Actinobacteriota 7%
p__Planctomycetota 6%
p__Bacteroidota 6%
p__Patescibacteria 4%
p__Gemmatimonadota 3%
p__Nitrospirota 3%
p__Verrucomicrobiota 1%
26 more
d__Eukaryota 0.3%
d__Archaea 1%
(B)
rplB
1899 reads
d__Bacteria
p__Pseudomonadota
c__Gammaproteobacteria 42%
c__Zetaproteobacteria 4%
c__Alphaproteobacteria 0.2%
p__Chloroflexota 10%
p__Planctomycetota 8%
p__Actinomycetota 8%
p__Patescibacteria 7%
p__Bacteroidota 5%
p__Gemmatimonadota 4%
p__Desulfobacterota 2%
p__Nitrospirota 1%
31 more
d__Archaea 2%
rpsB
1927 reads
d__Bacteria
p__Pseudomonadota
c__Gammaproteobacteria 43%
c__Zetaproteobacteria 4%
c__Alphaproteobacteria 1%
p__Chloroflexota 11%
p__Planctomycetota 7%
p__Actinomycetota 7%
p__Paceibacteria 6%
p__Bacteroidota 5%
p__Gemmatimonadota 3%
p__Desulfobacterota 2%
p__Nitrospirota 2%
30 more
d__Archaea 3%
(C)
rplB
BMS3A
1.86 mbsf
Archaean site
2242 reads
d__Bacteria
d__Archaea
p__Nitrospirota 34%
p__Desulfobacterota 5%
p__Bacteroidota 7%
p__Patescibacteria 8%
p__Planctomycetota 4%
p__JdFR-76 0.5%
p__Actinomycetota 5%
p__Camylobacterota 0.1%
p__Omnitrophota 3%
32 more
p__Pseudomonadota
c__Alphaproteobacteria 0.3%
c__Gammaproteobacteria 15%
p__Nanoarchaeota 8%
p__Hydrothermarchaeota 1%
6 more
rpsB
2262 reads
d__Bacteria
d__Archaea
p__Nitrospirota 33%
p__Desulfobacterota 5%
p__Bacteroidota 6%
p__Patescibacteria 8%
p__Planctomycetota 3%
p__JdFR-76 1%
p__Actinomycetota 6%
p__Camylobacterota 0.1%
p__Omnitrophota 2%
29 more
p__Pseudomonadota
c__Alphaproteobacteria 0.4%
c__Gammaproteobacteria 15%
p__Nanoarchaeota 9%
p__Hydrothermarchaeota 0.7%
7 more
(D)
rplB
BMS3B
1.96 mbsf
Archaean site
1725 reads
d__Bacteria
d__Archaea
p__Nitrospirota 24%
p__Desulfobacterota 7%
p__Bacteroidota 7%
p__Patescibacteria 6%
p__Planctomycetota 3%
p__JdFR-76 3%
p__Actinomycetota 2%
p__Camylobacterota 1%
p__Omnitrophota
38 more
p__Pseudomonadota
c__Alphaproteobacteria 3%
c__Gammaproteobacteria 24%
p__Nanoarchaeota 5%
p__Hydrothermarchaeota 2%
7 more
d__Euryarchaeota 0.07%
rpsB
1746 reads
d__Bacteria
d__Archaea
p__Nitrospirota 25%
p__Desulfobacterota 8%
p__Bacteroidota 5%
p__Patescibacteria 5%
p__Planctomycetota 4%
p__JdFR-76 3%
p__Actinomycetota 3%
p__Camylobacterota 0.9%
p__Omnitrophota 1%
37 more
p__Pseudomonadota
c__Alphaproteobacteria 3%
c__Gammaproteobacteria 22%
p__Nanoarchaeota 4%
p__Hydrothermarchaeota 2%
5 more

**Fig. S2**

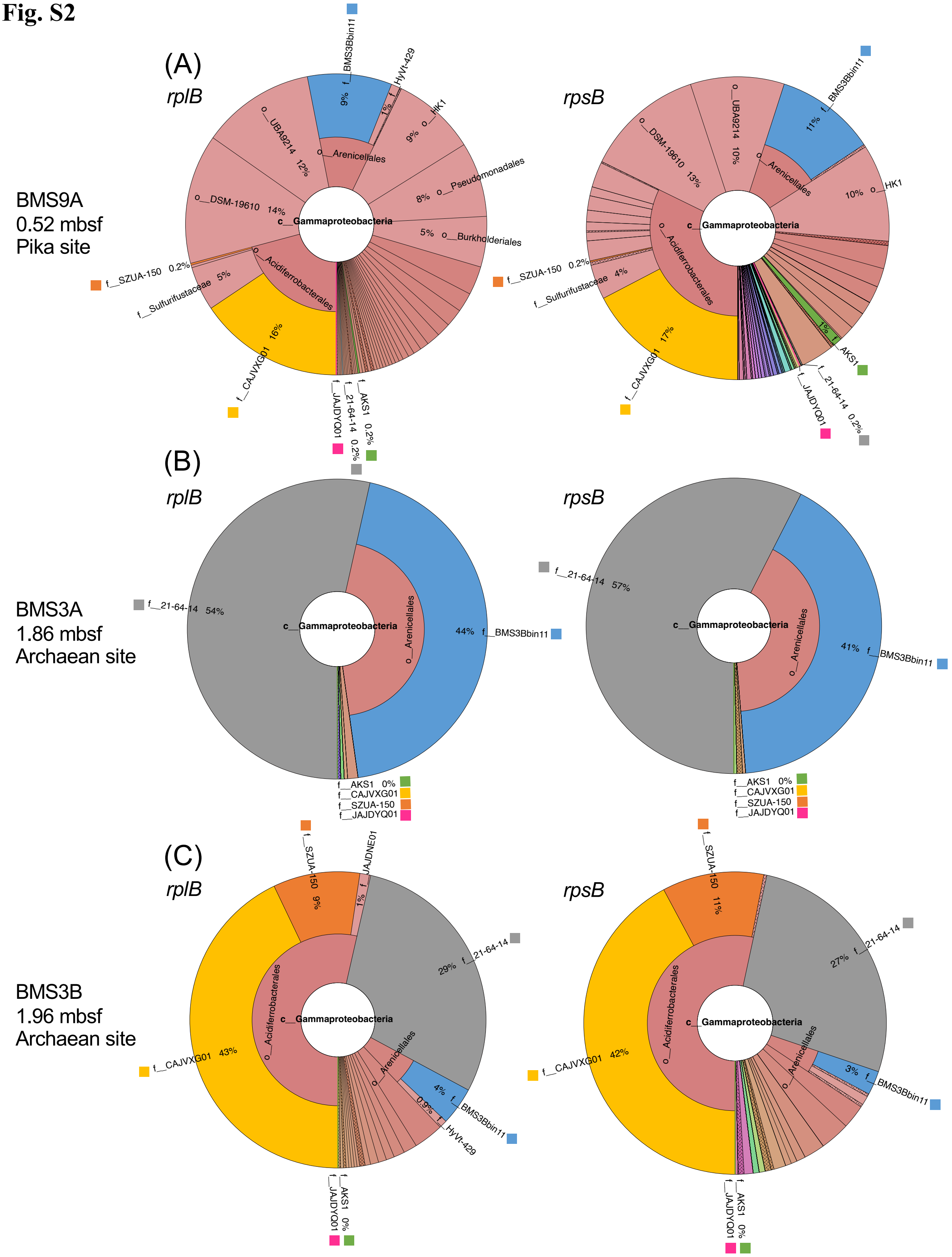

(A)
rplB
rpsB
BMS9A
0.52 mbsf
Pika site
c__Gammaproteobacteria
o__Arenicellales
o__Acidiferrobacterales
o__UBA9214 12%
o__DSM-19610 14%
o__HK1 9%
o__Pseudomonadales 8%
o__Burkholderiales 5%
f__BMS3Bbin11 9%
f__HyVt-429 1%
f__SZUA-150 0.2%
f__Sulfurifustaceae 5%
f__CAJVXG01 16%
f__JAJDYQ01
f__21-64-14 0.2%
f__AKS1 0.2%
o__UBA9214 10%
o__DSM-19610 13%
f__BMS3Bbin11 11%
o__HK1 10%
f__SZUA-150 0.2%
f__Sulfurifustaceae 4%
f__CAJVXG01 17%
f__AKS1 1%
f__21-64-14 0.2%
f__JAJDYQ01
(B)
rplB
rpsB
BMS3A
1.86 mbsf
Archaean site
c__Gammaproteobacteria
o__Arenicellales
f__21-64-14 54%
f__BMS3Bbin11 44%
f__AKS1 0%
f__CAJVXG01
f__SZUA-150
f__JAJDYQ01
f__21-64-14 57%
f__BMS3Bbin11 41%
f__AKS1 0%
f__CAJVXG01
f__SZUA-150
f__JAJDYQ01
(C)
rplB
rpsB
BMS3B
1.96 mbsf
Archaean site
c__Gammaproteobacteria
o__Acidiferrobacterales
o__Arenicellales
f__SZUA-150 9%
f__JAJDNE01 1%
f__21-64-14 29%
f__CAJVXG01 43%
f__BMS3Bbin11 4%
f__HyVt-429 0.9%
f__JAJDYQ01
f__AKS1 0%
f__SZUA-150 11%
f__21-64-14 27%
f__CAJVXG01 42%
f__BMS3Bbin11 3%
f__JAJDYQ01
f__AKS1 0%

**Fig. S3**

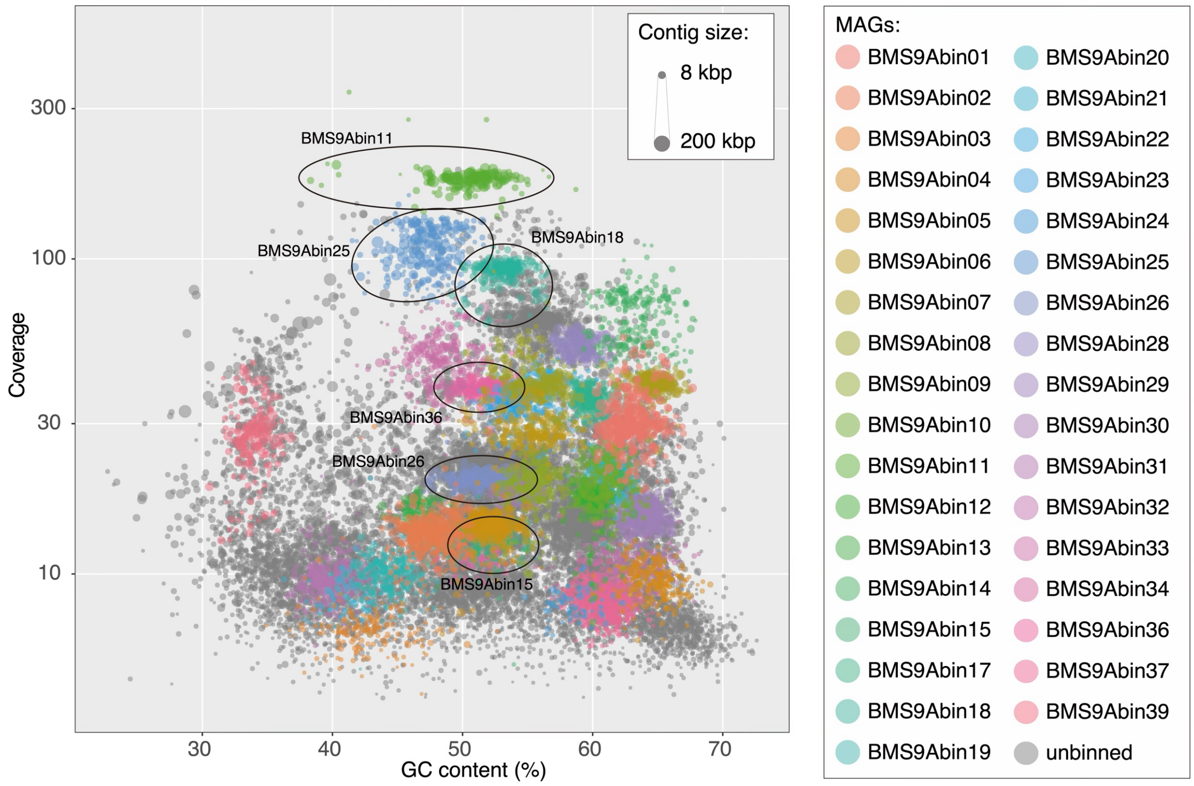
Contig size:
8 kbp
200 kbp
BMS9Abin11
BMS9Abin25
BMS9Abin18
BMS9Abin36
BMS9Abin26
BMS9Abin15
300
100
30
10
Coverage
30
40
50
60
70
GC content (%)
MAGs:
BMS9Abin01
BMS9Abin02
BMS9Abin03
BMS9Abin04
BMS9Abin05
BMS9Abin06
BMS9Abin07
BMS9Abin08
BMS9Abin09
BMS9Abin10
BMS9Abin11
BMS9Abin12
BMS9Abin13
BMS9Abin14
BMS9Abin15
BMS9Abin17
BMS9Abin18
BMS9Abin19
BMS9Abin20
BMS9Abin21
BMS9Abin22
BMS9Abin23
BMS9Abin24
BMS9Abin25
BMS9Abin26
BMS9Abin28
BMS9Abin29
BMS9Abin30
BMS9Abin31
BMS9Abin32
BMS9Abin33
BMS9Abin34
BMS9Abin36
BMS9Abin37
BMS9Abin39
unbinned

# Fig. S4

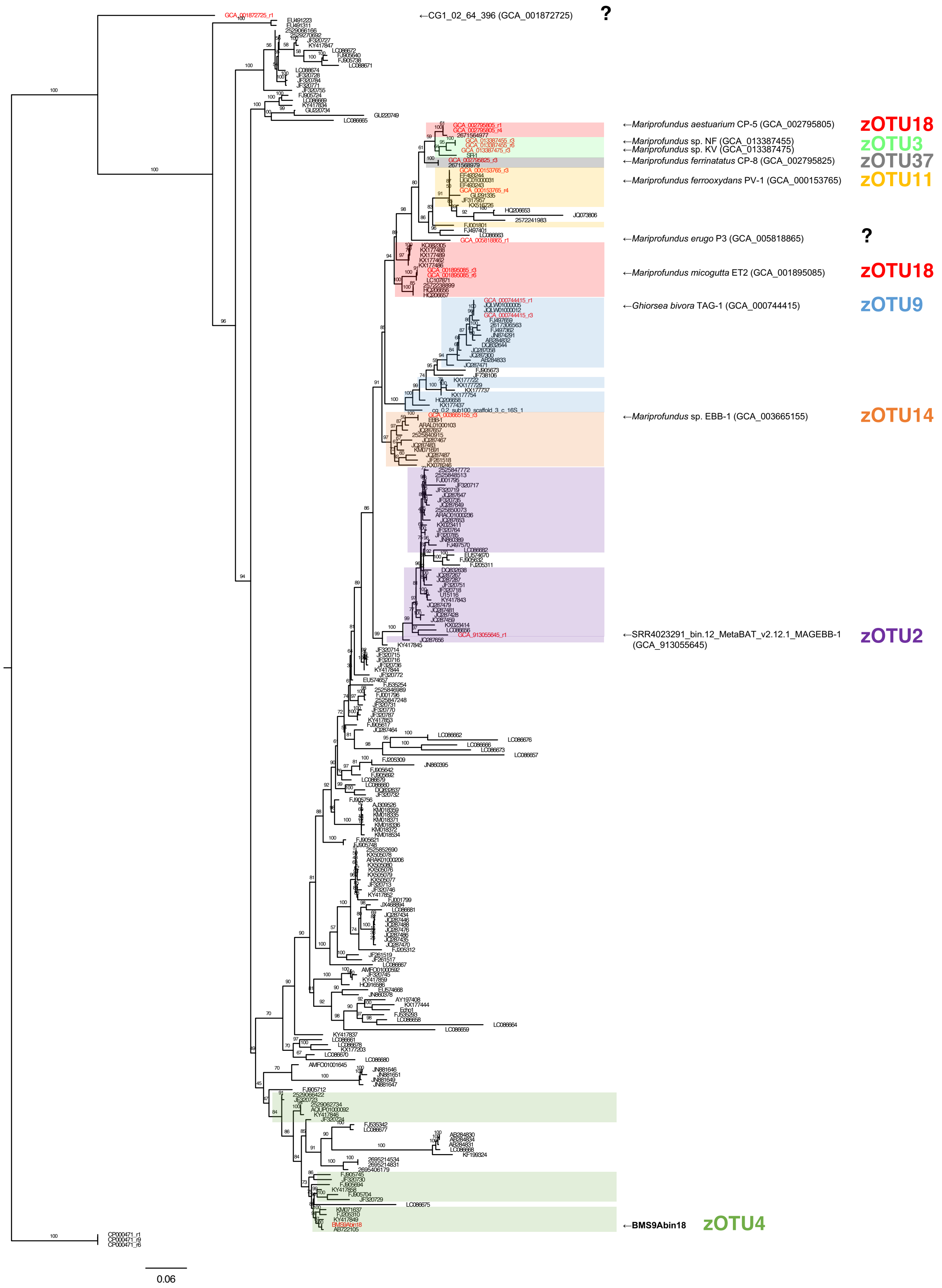

←CG1_02_64_396 (GCA_001872725)
?
←*Mariprofundus aestuarium* CP-5 (GCA_002795805)
zOTU18
←*Mariprofundus* sp. NF (GCA_013387455)
←*Mariprofundus* sp. KV (GCA_013387475)
zOTU3
←*Mariprofundus ferrinatatus* CP-8 (GCA_002795825)
zOTU37
←*Mariprofundus ferrooxydans* PV-1 (GCA_000153765)
zOTU11
←*Mariprofundus erugo* P3 (GCA_005818865)
?
←*Mariprofundus micogutta* ET2 (GCA_001895085)
zOTU18
←*Ghiorsea bivora* TAG-1 (GCA_000744415)
zOTU9
←*Mariprofundus* sp. EBB-1 (GCA_003665155)
zOTU14
←SRR4023291_bin.12_MetaBAT_v2.12.1_MAGEBB-1 (GCA_913055645)
zOTU2
←BMS9Abin18
zOTU4
0.06
